# Estimating fossil carbon contributions from chemicals and microplastics in Sweden's urban wastewater systems: A model-based approach

**DOI:** 10.1016/j.heliyon.2024.e37665

**Published:** 2024-09-12

**Authors:** Efstathios Reppas-Chrysovitsinos, Magdalena Svanström, Gregory Peters

**Affiliations:** Division of Environmental Systems Analysis, Department of Technology Management and Economics, Chalmers University of Technology, Gothenburg, SE412 96, Sweden

## Abstract

The importance of including fossil carbon in greenhouse gas emission assessments from wastewater treatment plants (WWTPs) is highlighted in the 2019 Intergovernmental Panel for Climate Change (IPCC) guidelines revision and underpinned by an increasing number of experimental studies. The present study introduces a model-based approach to estimate fossil carbon flows within Sweden's urban wastewater system, employing data on chemical and polymeric material flows as a starting point. Our findings show that fossil carbon constitutes approximately 12–17 % of the total carbon emissions to sewer systems. This result aligns with experimental data, which shows fossil carbon contributions to WWTP influents ranging from 4 to 28 %. Our analysis further indicates that microplastics contribute about 13 % of the fossil carbon influx to Swedish WWTPs, while organic chemicals account for the remaining 83 %.

## 1 Introduction and problem statement

Chemical industry products - chemicals and polymeric materials - are at the core of modern life. Electronics, textiles, pharmaceuticals and personal care products, food and feed, building materials, energy - virtually every industrial sector is underpinned by chemical production [1]. Chemical industry production is largely based on petroleum and other fossil resources for carbon feedstock and process energy [2]. The vast majority of the chemical products flowing through the technosphere every year therefore contain fossil carbon [2] and the growing carbon feedstock requirements for chemical production are turning the chemical industry into the sector with the fastest growing oil demand [3].

The wastewater system is one of the main pathways through which dissolved chemicals and suspended polymeric materials enter the environment. In general, the carbon found in wastewater is considered to be of biogenic origin and thus the CO_2_ emissions from wastewater treatment, including downstream emissions from effluent water and sludge, are typically considered carbon neutral [4]. However, experimental studies based on carbon isotope analysis showed that 4–28 % of the carbon entering wastewater treatment plants (WWTPs) could be of fossil origin and suggested that synthetic chemicals and polymers are the source of this fossil carbon in WWTPs [[5], [6], [7], [8]].

More specifically, the first study to measure fossil carbon inputs in U.S. WWTPs concluded that 25 % of the dissolved organic carbon in raw wastewater is of fossil origin and proposed that its source was petroleum-based household products [5]. A study investigating the fate of fossil carbon in Australian WWTPs collected and analyzed samples from four major WWTPs and found that fossil carbon constituted 8–14 % of the total organic carbon in the WWTPs that processed combined industrial and residential wastewater and 4–7% in the WWTPs receiving solely residential wastewater [6]. Another study focusing on the relative contribution of fossil carbon to total GHG emissions from WWTPs, investigated greenhouse gas (GHG) emissions for different wastewater treatment methods, and for different steps of the treatment process [7]. The study analyzed data from four WWTPs, three in the U.S. and one in Canada, and concluded that i) fossil carbon could amount to 28 % of the total carbon in primary influent, and ii) that the inclusion of non-biogenic CO_2_ in GHG accounting of WWTPs could result in a 23 % increase in the total GHG emissions of WWTPs, indicating that the IPCC accounting methodologies at the time were overlooking the presence of fossil carbon in raw wastewater. Based on these findings, the Intergovernmental Panel for Climate Change (IPCC) issued revised guidance to improve GHG emission estimates from WWTPs, encouraging the assessment of fossil carbon emissions [9]. The IPCC plays a central role in the global effort to understand and manage GHG emissions by setting guidelines for national GHG inventories, standardizing measurement methods and practices, delivering assessment reports on emissions sources and mitigation strategies, suggesting emission targets, and coordinating research efforts. Following the 2019 IPCC revisions, a study examining the fate of fossil and biogenic carbon in two Japanese WWTPs, each employing a distinct sludge treatment process, found no seasonal variation between autumn and winter in fossil carbon fate and distribution among air, water and sludge [8]. Significantly, when GHG emissions from sludge treatment were evaluated using the revised IPCC guidelines, they were found to be 36–65 % higher compared to assessments made under the original guidelines, highlighting the critical impact of accounting for fossil carbon in GHG emission estimates from WWTPs.

### 1.1 Objectives

Annual reporting for the urban water sector in Sweden is currently operating under the assumption that all the carbon in WWTPs is biogenic. Therefore, there are fossil GHG emissions from the urban water systems in Sweden that are not accounted for by the current national GHG-reporting practices. Expensive carbon-14 isotope analysis is the only practical experimental method for the determination of the fossil/biogenic carbon ratio. The basis of this method is the fact that fossil carbon is free of the radioactive ^14^C, as it has a half-life of 5,730 years. This study aims to provide a model-based estimation of the annual average fossil carbon inputs to Swedish WWTPs to support estimation of annual fossil GHG. To this end, the study builds upon an existing national-level model of chemical inputs to Swedish WWTPs [10], reported estimates of microplastics inflows to Swedish WWTPs, and desktop calculations of fossil carbon emissions from these chemicals and microplastics. Our approach is expected to be particularly useful for organizations that need an accounting approach, e.g. because they have set a target for carbon neutrality by 2030, such as Swedish Water [11] - the collaboration platform for the water sector in Sweden. To the best of our knowledge, there is a lack of model-based attempts to predict the fossil carbon inputs to WWTPs. Therefore, the overall objective of this study is to present a desktop method to account for non-biogenic carbon inflows to wastewater, to identify the potential significance of these emissions for reporting protocols and inform sustainable development strategies for urban water systems in Sweden.

## 2 Materials and methods

In principle, the information and knowledge required to estimate fossil carbon flows in wastewater from chemicals and microplastics include i) “bottom up” data on the identity and amounts of chemicals and polymers flowing through the technosphere into the wastewater treatment, ii) indications of whether the carbon used for their synthesis is of fossil origin or biogenic, iii) the physicochemical properties and the environmental processes that determine their transport and fate and, iv) degradation pathways that emit CO_2_.

Potential sources of fossil carbon in the raw wastewater of the combined wastewater system of Sweden include households, stormwater and any industrial wastewater that is not processed by industry. In brief, our approach brings together i) a national-level chemical emissions model for Swedish WWTPs [10], ii) reported estimates of microplastics emissions directly to Swedish WWTPs and to WWTP sources such as stormwater, and iii) data from various WWTPs of Sweden for industrial wastewater loads, treatment practices that make use of fossil carbon products, and total carbon inflows. Table 1 summarizes the materials – models and data – and estimation methods employed in this study, along with the main assumptions and limitations.

**Table 1:** Overview of data, estimation methods used, main assumptions and limitations.

**Source**	**Materials (models and data)**	**Fossil carbon estimation method**	**Assumptions**	**Limitations**
**Domestic**				
Household chemicals	Established national-level chemical emissions model providing annual inflow estimates to Swedish WWTPs for 2,007 substances [10]	Chemical flows [mass/year] × Carbon content calculated from chemical structure [% mass]	All chemicals are assumed to be of fossil origin	Does not account for biogenic chemicals
Household microplastics	Report providing emission estimates from Swedish households to sewer systems for synthetic polymers [12]	Microplastics flow estimate [mass/year] × Carbon content of polymeric materials (experimental data from literature, weighted average) [% mass]	Average carbon content from literature	Variability in polymer types and their carbon content
**Stormwater**				
Stormwater chemicals	Included in household emissions	Accounted in household emissions	Soluble stormwater fossil carbon contributions are negligible	May underestimate contributions from chemical long-range transport
Stormwater microplastics	Study providing emission estimates from tyre and bitumen wear to stormwater in Sweden [13]	Microplastics flow estimate [mass/year] × Carbon content of tyre wear particles (experimental data from literature) [% mass] × Stormwater fraction entering Swedish WWTPs (from literature) [%]	Uniform distribution of microplastics in stormwater	Potential geographic variability in stormwater microplastics
**Industrial loads**	Data on maximum permitted BOD_7_ loads for Gothenburg's WWTP [14], data on person equivalents (PE) capacity for the municipal WWTPs of Sweden [15], and TOC/BOD conversion factors [16]	BOD inflows to Gothenburg extrapolated to Sweden, based on Gothenburg's PE share −17 % of the total PE inflows in municipal WWTPs. Converted PE to BOD, following 1 PE = 70 g BOD/person·day [17]. Converted BOD to TOC, assuming TOC = 0.72 × BOD	Similar BOD and TOC conversion factors across Sweden	Regional variability in industrial contributions
**WWTP additives**	Data on chemicals and polymers used in wastewater treatment processes in Gothenburg's WWTP [17]	Chemicals and polymers used [mass/year] × Carbon content calculated from structure [% mass]. Converted PE to BOD and extrapolated from Gothenburg to Sweden, as above	Additive use is consistent across all Swedish WWTPs	Variability in WWTP practices and chemical usage
**Total organic carbon**	Data on BOD inflows in municipal WWTPs of Sweden [18] and TOC/BOD conversion factors [16]	BOD inflows (arithmetic mean) × conversion factor for typical sewage.	Reported national yearly totals for BOD inflows are representative of typical conditions.	Averaging may smooth out year-to-year variations in BOD inflows

### 2.1 Household input - chemicals and microplastics

#### 2.1.1 Fossil carbon in chemicals from households – the chemical database

This section draws from a 2022 study by Gustavsson et al. [10], which describes the development of a national-level chemical emissions model. This model estimates amounts of chemicals entering the wastewater system of Sweden, which are released from consumer products, textiles, and pharmaceuticals. The study combined data from: i) the Substances in Preparations in the Nordic Countries (SPIN) database, which documents chemicals in the Swedish market; ii) SWEREA, which covers chemicals in textiles; and iii) the National Board of Health and Welfare (NBHW), which focuses on pharmaceuticals used in Sweden. Using this data, the study delivered a chemical database of 2,007 chemicals that flow through the Swedish technosphere, and that are anticipated to enter WWTPs during their lifecycle. This database contains information on the identity (including structure, name, and various identifiers), estimated amounts in WWTPs, and physicochemical properties of these chemicals. A brief overview of the database's construction, along with the database itself, can be found in the Supporting Information.

#### 2.1.2 Fossil carbon in microplastics from households

Another source of fossil carbon in urban wastewater systems is microplastics emitted from households. The emissions study by Gustavsson et al. [10] was devoted to estimating chemical flows to wastewater and did not include microplastics. Probabilistic material flow assessment [[19], [20], [21]] has been used to establish polymer-specific models to describe flows of macroplastics and microplastics in the technosphere in Switzerland, including emissions from commodity plastics to wastewater. Currently there are no microplastics emissions models for Sweden. A 2016 report by the Swedish Environmental Research Institute, IVL, estimated that, on average, emissions of synthetic polymers from Swedish households to sewer are approximately 1,154 tonnes annually [12]. The main source of microplastics in household wastewater are consumer goods (such as personal care products) and microplastics emitted from the washing of synthetic articles [12,22]. The distribution and frequency of occurrence of polymers in households may vary considerably over time and application, due to changes in consumer preferences, regulatory standards, and technological advancements. These products, from their manufacture to disposal, release microplastics, a process compounded by fluctuations in technological and market share shifts. As a result, the polymeric composition of the influent and effluent of WWTPs is highly variable [23].

Estimating the microplastic share of fossil carbon inflows to urban wastewater systems requires information on the carbon content of these polymers and microplastics emissions. Table 2 presents the experimentally determined average carbon content of various polymers as reported in literature [24] via elemental analysis for different plastics. Therefore, these values represent the presence of both i) polymerization impurities and byproducts, as well as ii) the use of various polymeric additives - such as fillers, colors, plasticizers - in commercial plastics.

**Table 2:** *Carbon content (% weight) of different polymeric materials (experimental data, averaged). This table is adapted from C. Smeaton, 2021* [24].

**Polymer**	**Carbon content (% weight)**
Polystyrene (PS)	90 %
Low- density polyethylene (LDPE)	80 %
High- density polyethylene (HDPE)	80 %
Polypropylene (PP)	80 %
Polycarbonate	70 %
Polyurethane (PU)	65 %
Nylon	65 %
Polyethylene terephthalate (PET)	60 %
Polyacrylate	50 %
Polyvinyl chloride (PVC)	45 %
Polyoxymethylene	45 %

Given the predominance of PET, LDPE, HDPE, PVC, PP, and PS and their collective higher carbon content average of 75 % [24], we opted for a conservative estimate of 70 % as a weighted average for the carbon content of all polymers. This assumption is grounded in the likelihood that these common plastics, due to their widespread use and disposal, skew the overall carbon content towards the higher end. Thus, we model the total carbon in microplastics entering WWTPs from household sources as 70 % of the mass of these microplastics.

### 2.2 Stormwater input

Stormwater is understood as a major source of environmental pollutants and microplastics [[25], [26], [27]]. A major source of microplastics in stormwater is the runoff from the road network that contains microplastics emitted from various exterior paints, road paints, vehicle tyres and other vehicle-related plastic debris, and asphalt. The total annual microplastics emissions from tyre and road wear (TWP) in Sweden is estimated at 8,700 tonnes [13]. The variability in the composition of microplastics in experimental studies and theoretical calculations was found to be related to seasonal variability and, more specifically, to the use of studded tyres during winter in Sweden [13,28]. A recent report on microplastics emitted from tyre and road wear by the Swedish National Road and Transport Research Institute (VTI) concluded that the use of studded tyres causes polymer-based bitumen to tear off from the asphalt and estimated this process to generate around 200 tonnes of microplastics every year - a small proportion of the TWP [28]. TWP are acknowledged to be the dominant type of microplastics identified in stormwater [13,25,28,29] and, based on elemental analysis, their average carbon content is 85 % [30]. For simplicity, in this study we estimated the total carbon in microplastics in stormwater as 85 % of the mass of microplastics emitted from vehicles, asphalt, road paints, exterior paints and (re)construction activities.

A report by the Swedish EPA indicates that in Sweden 13 % of the stormwater enters the urban wastewater system, on account of the use of combined sewers [31]. Further, a Nordic Council of Ministers’ working paper that discusses efforts to reduce the release of microplastics from tyre wear, estimates that approximately 8 % of urban road runoff in Sweden undergoes treatment [32]. Based on this, we scaled the estimated 8,700 tonnes annual microplastics emissions from tyre and road wear in Sweden down by a factor of 13 % to estimate the amount entering the WWTPs from stormwater.

### 2.3 Industrial input

Based on a report from Gryaab, the Gothenburg WWTP, industrial inputs for 2020 were 7 % of the total inflows [17]. To identify sources of fossil carbon in the industrial inflow, we contacted Gryaab to obtain information on the industrial biological oxygen demand (BOD_7_) loads from the respective industrial facility permits, for the companies that are active in the greater Gothenburg area and are connected to the local WWTP [14]. In total, there were at the time 14 such permits. Eight of these permits were for companies in the food and beverages industry, and two for the tobacco industry. The remaining four permits were, respectively, for a company in the pharmaceutical industry, a car manufacturing company, a company in the chemical industry, and a textile services company. The pharmaceuticals company permit was for both industrial and sanitary wastewater, while the car manufacturing company permit was only for industrial wastewater. We assumed that most of the carbon in the wastewater from the food and beverage companies is of biogenic origin and we therefore decided to exclude these eight permits from our calculations. Among the other six permits, the highest BOD_7_ load is 132.1 tonnes per year and the minimum load is 0.6 tonnes per year. The total load of these six permits is 275.35 BOD_7_ tonnes per year. We highlight that these values are for maximum permittable BOD_7_ loads - not the actual loads entering the WWTP.

In the absence of similar information for other WWTPs in Sweden, we decided to scale up these permits using data from the Statistical Database of Statistics Sweden according to which the inflows to the WWTP of Gothenburg are on average 17 % of the total inflows to Swedish WWTPs [15]. This assumption implies that the mix of industries in Gothenburg and the wastewater they produce and direct to municipal WWTPs are broadly representative of the national industrial distribution. We estimate that the annual BOD_7_ load from industry to municipal WWTPs in Sweden is ∼1,620 tonnes (calculated as 275.35 BOD_7_ tonnes per year divided by 17 %). We then estimated TOC from BOD, using a linear relationship (equation (1)) and recently published conversion factors [16]. For industrial wastewater, we opted for a conversion factor (*a*) equal to 0.72, as it reflects a balanced share of biodegradable and non-biodegradable organic matter, which is typical for many industrial discharges.

(1) TOC=a×BOD

### 2.4 Wastewater treatment additives

WWTPs typically use a number of fossil-based chemical products for the treatment of wastewater. Dominant among these are anionic and cationic polymers used to enhance settling processes, as well as carbon sources used in secondary treatment. Conversations with WWTP managers in Sweden indicated that carbon sources are sometimes of plant origin, particularly when molasses is used, but when methanol is used it is typically a fossil carbon product. Much smaller quantities of carbon-based chemicals are also used for cleaning and for other purposes in WWTPs. Here, we used the reported amounts of polymer used for precipitation, thickening, drainage, and post-sedimentation (450.3 tonnes) and methanol (2,436 tonnes) used in the WWTP of Gothenburg in 2020 [17] and extrapolated assuming that the WWTP of Gothenburg accounts for 17 % of the total BOD inflows in municipal WWTPs of Sweden, following data on reported BOD inflows from the Statistical Database of Statistics Sweden to estimate the relevant national amounts for Sweden [15].

### 2.5 Total organic carbon inputs

To estimate the total fossil and biogenic carbon inputs to Swedish wastewater systems we used reported data for BOD loads from the Statistical Database of Statistics Sweden [18]. Subsequently, to estimate TOC based on BOD, we employed equation (1) and recently published conversion factors [16]. For typical sewage, the conversion factor (*a*) ranges from 0.67 to 1.0.

## 3 Results

### 3.1 Total fossil carbon in urban wastewater systems in Sweden

Table 3 delivers the total estimated fossil carbon entering the urban wastewater treatment system in Sweden annually, along with the share of each category. In total, 24,693 tonnes of fossil carbon are estimated to enter Swedish WWTPs. Household chemicals account for 61 % of the total estimated amount and the combined microplastics account for 13 %. WWTP additives are the second largest carbon group after household chemicals. The industrial load of fossil carbon to municipal WWTPs is only ∼4.7 %.

**Table 3:** Total annual inflow of fossil carbon to urban wastewater systems in Sweden.

**Source**	**Carbon flow (tonnes/year)**
Domestic flows (total)	15,834
Household chemicals	15,026
Household microplastics	808
Stormwater flows (total)	961
Stormwater chemicals	Accounted in household emissions
Stormwater microplastics	961
Industrial load	1,169
WWTP additives (total)	6,729
methanol	5,383
polymers	1,346
**Total**	**24,693**

Of the household chemicals, Table 4 presents the top 25 chemicals that contribute 68 % (10,222 tonnes) of this carbon flow, with each of them having a flow higher than 100 tonnes/year. Interestingly, ethanol contributes 19 % of the fossil carbon flowing in the wastewater from households in Sweden. This is industrial ethanol and excludes alcohol in beverages, which is assumed to be biogenic. Based on these calculations, the top 10 chemicals combined contribute more than the stormwater microplastics, while the estimated carbon input from industrial ethanol alone is twice as high as the combined carbon input from the household microplastics and the industrial load.

**Table 4:** The carbon inflow from the top 25 chemicals in the database (10,222 tonnes/year) amounts to 68 % of the total carbon inflows from chemicals to wastewater treatment plants (15,026 tonnes/year). The "indicative consumer uses" column contains the name of the largest (by tonnage, for 2021 in Sweden) use category identified as relevant for household emissions to wastewater. The information on the categories is extracted from the “National use category” dataset of the SPIN database, for Sweden.

Name	CAS #	Molecular Formula	Emissions [t/y]	Total Carbon [t/y]	Indicative consumer uses
Ethanol	64175	C_2_H_6_O	5,528	2,882	solvent
1,2-Cyclohexanedicarboxylic acid, 1,2-diisononyl ester	166412788	C_26_H_48_O_4_	2,143	1,576	softeners for plastic, rubber, paint, and adhesive
EDTA	60004	C_10_H_12_N_2_O_8_	2,480	1,019	cleaning/washing agents
Isopropanol	67630	C_3_H_8_O	946	567	solvent
Petroleum distillates - heavy paraffinic, dewaxed solvent	64742650	C_20_H_12_	456	434	lubricant
Hydroxyformic acid	463796	CO_3_	2,134	413	confidential use
N-(4-aminophenyl)-4-formylbenzamide	24938645	C_14_H_12_N_2_O_2_	574	402	confidential use
2-Propanone	67641	C_3_H_6_O	424	263	solvent
1,2,3-propanetricarboxylic acid, 2-hydroxy-, monohydrate	5949291	C_6_H_8_O_7_	691	259	PH-regulating agents
Alcohols, C12-18, ethoxylated	68213230	C_24_H_50_O_6_	313	208	textile detergent
Sulfuric acid, monododecyl ester	151417	C_12_H_25_O_4_S	372	201	confidential use
1,2-Benzenedicarboxylic acid, bis(2-propylheptyl) ester	53306540	C_28_H_46_O_4_	259	195	filler/softener
Alcohols, C12-18, ethoxylated propoxylated	69227210	C_14_H_30_O	241	189	cleaning/washing agents for washing machines
2-Propanol, 1-methoxy-, acetate	108656	C_6_H_12_O_3_	333	182	solvent
1,2-Propanediol	57556	C_3_H_8_O_2_	381	181	textile detergent
Poly(oxy-1,2-ethanediyl), alpha-(2-propylheptyl)-omega-hydroxy-	160875661	C_12_H_26_O_2_	243	173	degreaser
1,2-Benzenedicarboxylic acid, diisononyl ester	28553120	C_26_H_42_O_4_	191	143	softeners for plastic, rubber, paint, and adhesive
Poly(oxy-1,2-ethanediyl), -tridecyl- -hydroxy-, branched	69011365	C_23_H_48_O_6_	198	130	textile detergent
Alcohols, C12-15, ethoxylated	68131395	C_22_H_46_O_6_	187	121	textile detergent
1,2,3-Propanetriol	56815	C_3_H_8_O_3_	303	119	cosmetics
Fatty acids, lanolin	68424431	C_18_H_34_O_2_	152	117	anti-corrosion materials
Ethaneperoxoic acid	79210	C_2_H_4_O_3_	364	115	textile detergent
Acetic acid, butyl ester	123864	C_6_H_12_O_2_	183	114	solvent
Glycerides, C16-18 mono- and di-	85251770	C_19_H_38_O_4_	163	112	emulsifier
Poly(oxy-1,2-ethanediyl),α-hydro-ω-hydroxy- Ethane-1,2-diol, ethoxylated	25322683	C_10_H_22_O_6_	216	109	cleaning/washing agents for washing machines
**Total emissions**			**19,475**	**10,222**	

Ethanol's relative dominance over other chemicals, household microplastics and the industrial load reflects its wide use as a solvent that is present in many consumer products, including personal care products, household cleaning products, pharmaceuticals, paints, coatings, inks, etc. as well as its role as a building block for chemicals [33]. Ethanol is approximately 52 % carbon by mass. Household microplastics are typically richer in carbon than ethanol (see Table 2), however, their overall estimated emissions are fairly low in comparison. The industrial load to municipal WWTPs is also relatively low in comparison, possibly reflecting that most industries treat their wastewater in-house and do not opt for the municipal WWTPs after the introduction of special rates for extra burdensome industrial loads by many municipalities in the recent years.

Based on the information available from the SPIN database, the chemicals in our study are used in a multitude of consumer goods, with applications varying over time. As of 2021, among the top 25 chemicals listed in Table 4, eight are used in washing/cleaning products, five as solvents, and three as softeners. Additionally, six other chemicals are each used for distinct purposes: lubrication, emulsification, cosmetics preparation, pH regulation, anti-corrosion, and degreasing. Finally, three chemicals have confidential uses. Excluding these confidential cases, the remaining categories may reasonably be expected to be emissions to wastewater.

### 3.2 Total organic carbon (TOC) entering the WWTPs in Sweden

Table 5 presents the total annual incoming BOD to the WWTPs of Sweden for every other year since 2014, as reported in the Statistical Database of Statistics Sweden [18]. We used the arithmetic mean of the reported data, 214,283 tonnes of BOD. This value, following equation (1), is equivalent to 143,569 tonnes of TOC(*a* *=* 0.67) to 214,283 tonnes of TOC (*a* = 1.0)and, consequently, the total estimated fossil carbon – 24,693 tonnes – accounts for approximately 12–17% of the TOC in the inflow of the Swedish WWTPs.

**Table 5:** Incoming BOD7 (organic compounds) to municipal wastewater treatment plants by every other year, data collected from Statistics Sweden [18].

**Year**	**Total incoming BOD [tonnes] - Statistical Database Sweden**
2014	208,340
2016	215,488
2018	224,533
2020	208,770

## 4 Discussion

### 4.1 Study highlights

We present a simple, model-based approach for estimating flows of fossil carbon to Swedish WWTPs. Our results (∼12–17 % of the total WWTP carbon inflows) are within the range of the limited experimental data previously reported in the literature (4–28 %) [[5], [6], [7], [8]]. This study is largely based on a published chemical emissions model that was established to investigate flows of toxic chemicals to the Swedish wastewater system [10]. Our method builds upon this emissions modelling and estimates of microplastics emissions to assess fossil carbon flows from chemicals and microplastics in the urban wastewater system of Sweden. We therefore see our approach as a useful step towards addressing the Swedish goals for a toxic-free environment, the 2030 Agenda for Sustainable Development Goals, and national targets for climate change.

### 4.2 Greenhouse gas emissions and environmental implications

Ignoring non-biogenic carbon, wastewater treatment is estimated to account for nearly 1.57 % of the total global greenhouse gas (GHG) emissions [34], while the CH_4_ and N_2_O emissions from WWTPs are estimated to represent approximately 5 % of the total non-CO_2_ GHG emissions [35]. In Sweden, the annual territorial emissions of GHGs are estimated at 48 million tonnes of carbon dioxide equivalents [36]. Assuming that our estimate of approximately 24,700 tonnes of fossil carbon inflows to Swedish WWTPs annually will in time completely break down to carbon dioxide, 90,650 tonnes of carbon dioxide will be emitted. Considering that the average passenger vehicle emits about 4.6 tonnes of CO_2_ per year [37], these 90,650 tonnes of CO_2_ are roughly equivalent to the annual emissions from about 19,715 passenger vehicles. This is a conservative estimation, not considering the temporarily enhanced climate impact that can be expected from the emission of other GHGs associated with the breakdown process, such as methane.

### 4.3 Interpretation of results

Compared to the aforementioned Swedish GHG emissions, these fossil-based CO_2_ emissions from the WWTPs of Sweden account for ∼0.19 % of the total fossil emissions. While this percentage may appear negligible in the face of the current challenges to reduce national fossil carbon emissions, it gains significance when compared to the emissions of the Swedish wastewater treatment (WWT) sector. According to data collected from the Swedish Environmental Protection Agency and reported by the Statistical Database of Statistics Sweden [38], the WWT sector emitted an average of 225,070 tonnes of CO_2_-eqv annually between 1990 and 2019, with an average annual reduction of approximately 1,020 tonnes of CO_2_-eqv. In this context, our estimate of 90,650 tonnes of CO_2_ represents 40 % of the sector's annual emissions and is approximately 89 times greater than the average annual reduction. Given the Swedish water sector's goal to achieve climate neutrality by 2030, these insights into fossil carbon sources, particularly from the degradation of chemicals and microplastics that may yield even stronger GHGs, are too significant to disregard.

Beyond identifying the presence of fossil carbon in Sweden's urban wastewater treatment system, our study marks a step towards understanding its sources. This opens up new avenues for identifying key stakeholders and formulating strategies within Swedish society to address this issue effectively. Potential strategies and technologies that could help reduce fossil carbon emissions to wastewater systems include waste minimization and recycling, opting for green solvents and bio-based alternatives to fossil-based chemicals and polymeric materials in the production phase, reducing carbon-intensive products when possible, improving storm-water management and reducing urban runoff, modifying wastewater treatment processing, as well as promoting consumer awareness.

### 4.4 Method limits and limitations

The main source of fossil carbon data in this assessment is the international SPIN chemicals database and, thus, any of the emission modelling uncertainties pertaining to the database are expected to influence the results of this study. One of our main assumptions is that all the chemicals in the database and all polymeric materials are made of fossil carbon. This assumption is not expected to be a major weakness for our method given that, until 2019, the total production volume of bio-chemicals and biopolymers was less than 1 % of the total production volume of fossil-based chemical products in Europe [39]. The chemical database does not include pesticides or other fossil-based substances for agricultural use. However, even though pesticides can be found in the sludge of Swedish WWTPs [40], they typically occur in relatively low concentrations compared to other chemical groups [41]. We therefore do not consider the absence of pesticides as a major drawback of our approach.

The emissions of pharmaceuticals in the Gustavsson database are estimated using data on metabolism [10]. In the context of aiming to assess emissions of toxic substances, metabolic pathways that lead to the elimination of the parent pharmaceutical compounds and, consequently, to the excretion of-typically less toxic-metabolites may be of importance. However, the carbon in the metabolites of fossil-based pharmaceuticals excreted to the wastewater will also be of fossil origin. We assumed that the parent compounds and their metabolites are excreted renally and/or biliary and that pulmonary excretion and other losses such as sweat, saliva, and breast milk are negligible. We therefore proceeded with the emission estimates originally proposed in the Gustavsson study.

The dominant pathway to urban wastewater for the substances considered in the chemical database is household discharges, while the Mackay “level-1” steady-state model employed in the creation of the database (see Supporting Information) accounts for chemical losses to other environmental compartments. Our model assumes that all substances in the chemical database arrive in household or industrial wastewater, while stormwater supplies most of the microplastics. Although the carbon from these stormwater microplastics is a marginal contribution to the total fossil carbon flow in Swedish WWTPs, this is not due to a lack of microplastics in the environment. A recent report for Gothenburg's municipal WWTP highlighted that stormwater, along with drainage water and inflow and infiltration, makes up 60 % of the total wastewater influent water [17]. If we assumed that all stormwater entered municipal WWTPs, its microplastics would account for 7,395 tonnes of fossil carbon. This would make stormwater the second largest source of fossil carbon, increasing the estimated fossil carbon share to 15–22 % of the total WWTP carbon inflows.

Our model does not account for any non-local sources, such as the atmospheric deposition of fossil-based chemicals and microplastics in Sweden that were released elsewhere. Due to its northern latitude, Sweden is affected by the deposition of organic pollutants with high long-range transport potential [42,43], as well as by contamination from atmospheric particles that contain fossil carbon and atmospheric elemental carbon of fossil origin, from neighboring regions [44]. Moreover, it is known that microplastics are capable of atmospheric transport and deposition in remote regions [45,46] and that Scandinavia is one of the regions with the highest microplastic deposition estimates [46]. A study focused on the greater Paris area in France (2,500 km^2^) estimated the annual atmospheric deposition of fibers between 3 and 10 tonnes, with 29 % of these fibers being made from petrochemicals [47]. While these pathways are interesting, they are minor compared to our estimate of ∼24,693 tonnes of fossil carbon entering Swedish WWTPs annually. Thus, we are confident that omitting non-local sources of fossil carbon does not compromise our model's robustness.

Considering the assumptions invoked in scaling up from regional to national levels, the estimated fossil carbon share of the industrial activities that enters the combined urban wastewater system of Sweden is the most uncertain one. However, industrial development is widespread and tends to coincide with major population centers in Sweden, so scaling up by urban wastewater flows is reasonable. Additionally, since the estimated contribution of industrial flows is less than 5 % of the total flows, doubling or halving this amount does not change the scale of the total estimate greatly.

The assumption that all chemicals and polymers eventually break down into CO_2_ is meaningful for most chemical products. Experimental studies have shown that certain polymers, such as polystyrene, polyethylene and polypropylene, can degrade to carbon dioxide and dissolved organic carbon on decadal time scales [[48], [49], [50]]. The fossil carbon in synthetic chemicals and microplastics released in the environment via WWTPs may function as an energy source for certain microbes in the receiving aquatic environments, producing both carbon dioxide and methane [51]. However, highly persistent chemicals - such as certain PFAS - are not expected to degrade in the environment, even within geological timescales [52]. The estimated amounts of such persistent chemicals in our study were orders of magnitude lower than most chemicals and therefore their presence does not influence the overall assessment. Nonetheless, highly persistent chemicals associated with undesired effects on humans and ecosystems constitute acute environmental challenges [53,54] that warrant further study alongside climate change and changes in the carbon cycle [[55], [56], [57]].

Our goal was to develop a model-based method for estimating fossil carbon inflows into Sweden's urban wastewater system. Confronted with significant data availability challenges, our study shifted towards a methodology designed to navigate these constraints. This resulted in a preliminary estimation model, utilizing: i) a national-level emissions model for organic chemicals in Swedish wastewater, ii) national assessments of microplastics emissions, iii) comprehensive data on Sweden's wastewater treatment infrastructure, iv) specific data on wastewater treatment practices in Gothenburg, and v) local data on industrial contributions to Gothenburg's WWTP.

Our approach integrates chemical and polymeric material emissions to Swedish WWTPs from various sources, making a historical analysis challenging due to diverse, limited, and low-resolution data. This is especially true for fossil carbon inputs from industrial loads, WWTP additives, and polymeric materials from households and stormwater. Although feasible for the household chemical emissions model based on SPIN data, we chose not to explore historical trends to focus on introducing our method first.

### 4.5 Study contributions

Our study introduces a novel model-based, desktop method for assessing fossil carbon flows into WWTPs, representing an advancement beyond existing experimental methods, while also highlighting the limitations of current models and data in performing this task. Compared to experimental and field studies [[5], [6], [7]], the main limitations of our approach are that currently it cannot be used to account for seasonality (when carbon flows are higher), locality (how carbon flows vary within Sweden) and the breadth of WWTP technologies (how the technologies employed affect the fate of carbon flows).

However, considering the vast expense associated with carbon isotope analysis to assess fossil carbon inflows to WWTPs, we believe that a desktop approach like ours can be used in combination with future experimental and field studies to improve GHG accounting by elucidating fossil carbon sources to WWTPs, identifying fossil carbon hotspots in the urban water treatment systems, and filling data gaps. While our findings shed light on the limitations and constraints of our approach, they also underscore the need for further research and methodological advancements.

### 4.6 Future perspectives

Our reliance on the SPIN database means that this approach is directly applicable to the three other Nordic countries which are also covered by SPIN (Denmark, Norway, and Finland) and we expect that it could also be applied in countries and regions with similar chemical inventories [58]. For fossil carbon inputs to municipal WWTPs that come from industrial loads, WWTP additives and polymeric materials from households, our method is based on simple extrapolations and is therefore more readily applicable where sufficient data exists.

Our method could benefit from improvements in the currently disappointing data landscape for chemical emissions. In this direction, we believe that improving the “Exposure Index” (EI) screening-level exposure tool of the SPIN database [59] to better identify substances that are likely to end up in a sewage treatment plant (STP) would be highly beneficial. Currently, the EI tool is mainly based upon expert judgement for direct chemical emissions, which are in turn based on consumer use patterns, and does not take into consideration the physicochemical properties of a substance. A 2011 study by Undeman et al. that evaluated the EI for estimating relative emissions of chemicals to STPs against measured data from WWTPs concluded that the EI tool requires substantial improvements and provided recommendations to improve the tool [60]. Even though the EI tool has been updated since the aforementioned evaluation, our initial attempt to use it to systematically reduce uncertainties based on usage information was not successful. Mining the SPIN database to create a list of substances that circulate in the Swedish market with high EI for STPs, resulted in a lengthy list of very high-volume chemicals such as fuels, petrochemicals, and building-block chemicals. Due to their multitude and volume, these substances dominated our mining exercise. Our preliminary assessment indicated that the inclusion of these chemicals would lead to considerable overestimation of the carbon flowing through the technosphere, mainly by accounting multiple times for carbon in substances of different life cycle phases - for example, both in the parent compound and the final product.

Our approach could further benefit from more detailed statistical data on the industrial loads that enter the municipal WWTPs in Sweden and/or up-to-date information on the spatial distribution of the national industrial activity. The former would be a valuable addition to the existing wealth of data that is publicly available through the Statistical Database of Statistics Sweden, while the latter could be a follow-up to the 2002 statistical inventory of industrial clusters in Sweden [61]. This additional information could prove crucial for a deeper exploration of the roles various stakeholders play and for pinpointing synergies crucial for effective mitigation strategies.

A modeled WWTP approach could serve as a starting point for a mass-balance assessment of carbon fate. SimpleTreat is such a model that assesses chemical fate in WWTPs that also partly accounts for the role of technology on chemical fate [62]. However, such tools are typically designed for individual chemicals rather than polymers and assess one chemical at a time. Therefore, these models are not readily applicable to studies aiming to estimate fossil carbon flows from polymeric materials and thousands of chemicals.

The preliminary estimates and method presented in this study could be extended to inform carbon management strategies from a life cycle perspective by including mass balance calculations to assess the distribution of fossil carbon in the products and by-products of WWTPs. Such a framework is particularly valuable for research aimed at enhancing carbon recycling through the exploration of wastewater treatment pathways equipped with carbon capture and utilization (CCU) capabilities [63]. Moreover, it facilitates a more precise integration of these pathways with broader studies assessing the viability of CCU technologies in fulfilling the chemical industry's carbon requirements, both as feedstock and for operational energy [64].

## 5 Conclusion

We have developed a model-based method for estimating fossil carbon inflows into Sweden's urban wastewater system. The results, ranging from 12 to 17 % of the total WWTP carbon inflows, align with limited experimental data reported in the literature. This approach is based on a chemical emissions model initially designed to investigate toxic chemical flows into the Swedish wastewater system and incorporates estimates of microplastics emissions from households and stormwater, data on industrial loads and chemicals and polymers used in wastewater treatment processes.

Our study underscores the importance of addressing fossil carbon emissions in wastewater systems and provides a cost-effective alternative to carbon isotope analysis that can complement future experimental studies. With further improvements in chemical and polymer emissions modelling and more high-resolution data, our approach can be refined to offer more precise and comprehensive insights into fossil carbon flows and contribute significantly to climate neutrality goals.

## Role of the funding source

We hereby declare that the funding source had no involvement in study design; in the collection, analysis and interpretation of data; in the writing of the report; and in the decision to submit the article for publication.

## CRediT authorship contribution statement

**Efstathios Reppas-Chrysovitsinos:** Writing – review & editing, Writing – original draft, Visualization, Validation, Methodology, Investigation, Formal analysis, Data curation, Conceptualization. **Magdalena Svanström:** Writing – review & editing, Validation, Methodology, Investigation, Funding acquisition, Formal analysis, Data curation, Conceptualization. **Gregory Peters:** Writing – review & editing, Validation, Methodology, Investigation, Funding acquisition, Formal analysis, Data curation, Conceptualization.

## Declaration of competing interest

The authors declare that they have no known competing financial interests or personal relationships that could have appeared to influence the work reported in this paper.

## References

[1] Cefic (2014). Landscape of the European chemical industry. http://www.cefic.org/Documents/Landscape-European-chemical-industry/Landscape-of-the-European-Chemical-Industry-March-2014.pdf.

[2] International Energy Agency (2017). Tracking clean energy progress. http://www.iea.org/etp/tracking.

[3] International Energy Agency (2018). The future of petrochemicals: towards more sustainable plastics and fertilisers. https://www.iea.org/reports/the-future-of-petrochemicals.

[4] Peters G.M., Lundie S. (2001). Life-cycle assessment of biosolids processing options. J. Ind. Ecol..

[5] Griffith D.R., Barnes R.T., Raymond P.A. (2009). Inputs of fossil carbon from wastewater treatment plants to U.S. Rivers and oceans. Environ. Sci. Technol..

[6] Law Y., Jacobsen G.E., Smith A.M., Yuan Z., Lant P. (2013). Fossil organic carbon in wastewater and its fate in treatment plants. Water Res..

[7] Tseng L.Y., Robinson A.K., Zhang X., Xu X., Southon J., Hamilton A.J., Sobhani R., Stenstrom M.K., Rosso D. (2016). Identification of preferential paths of fossil carbon within water resource recovery facilities via radiocarbon analysis. Environ. Sci. Technol..

[8] Liu C., Oshita K., Takaoka M., Fukutani S. (2021). Behaviour of fossil and biogenic carbon in sewage sludge treatment processes and their impacts on greenhouse gas emissions. Chem. Eng. Trans..

[9] Bartram D., Short M.D., Ebie Y., Farkaš J., Gueguen C., Peters G.M., Zanzottera N.M., Karthik M. (2019).

[10] Gustavsson M., Molander S., Backhaus T., Kristiansson E. (2022). Estimating the release of chemical substances from consumer products, textiles and pharmaceuticals to wastewater. Chemosphere.

[11] Vatten Svenskt (2023). Tillsammans för en klimatneutral VA-bransch. https://www.svensktvatten.se/medlemsservice/klimatneutral-va/.

[12] Magnusson K., Eliasson K., Fråne A., Haikonen K., Hultén J., Olshammar M., Stadmark J., Voisin A. (2016).

[13] Järlskog I., Strömvall A.M., Magnusson K., Gustafsson M., Polukarova M., Galfi H., Aronsson M., Andersson-Sköld Y. (2020). Occurrence of tire and bitumen wear microplastics on urban streets and in sweepsand and washwater. Sci. Total Environ..

[14] Davidsson F. (2019). PM - industriell BOD-belastning kopplat till Länsstyrelsens förslag till omfattning av tillståndet för Ryaverket. https://www.gryaab.se/wp-content/uploads/2019/06/Bilaga-7-Industribelastning.pdf.

[15] Dimberg A. (2022). Capacity (pe) by region/size, treatment methods and every other year. Stat. Database..

[16] la Cour Jansen J., Arvin E., Henze M., Harremoës P. (2021). Polyteknisk Forlag.

[17] Videbris K.-E. (2021).

[18] Dimberg A. (2022). Municipal wastewater treatment plants: incoming and outgoing amounts and treatment efficiency. Every other year 2014 - 2020, Stat. Database.

[19] Kawecki D., Nowack B. (2019). Polymer-specific modeling of the environmental emissions of seven commodity plastics as macro- and microplastics. Environ. Sci. Technol..

[20] Liu Z., Nowack B. (2022). Probabilistic material flow analysis and emissions modeling for five commodity plastics (PUR, ABS, PA, PC, and PMMA) as macroplastics and microplastics. Resour. Conserv. Recycl..

[21] Mennekes D., Nowack B. (2023). Predicting microplastic masses in river networks with high spatial resolution at country level. Nat. Water..

[22] Naturvårdsverket (2021). Microplastics in the environment 2019. https://www.naturvardsverket.se/om-oss/publikationer/6900/microplastics-in-the-environment-2019/.

[23] Okoffo E.D., O'Brien S., O'Brien J.W., Tscharke B.J., Thomas K.V. (2019). Wastewater treatment plants as a source of plastics in the environment: a review of occurrence, methods for identification, quantification and fate. Environ. Sci. Water Res. Technol..

[24] Smeaton C. (2021). Augmentation of global marine sedimentary carbon storage in the age of plastic, Limnol. Oceanogr. Lett..

[25] Werbowski L.M., Gilbreath A.N., Munno K., Zhu X., Grbic J., Wu T., Sutton R., Sedlak M.D., Deshpande A.D., Rochman C.M. (2021). Urban stormwater runoff: a major pathway for anthropogenic particles, black rubbery fragments, and other types of microplastics to urban receiving waters. ACS ES&T Water.

[26] Hall K.J., Anderson B.C. (1988). The toxicity and chemical composition of urban stormwater runoff. Can. J. Civ. Eng..

[27] Fairbairn D.J., Elliott S.M., Kiesling R.L., Schoenfuss H.L., Ferrey M.L., Westerhoff B.M. (2018). Contaminants of emerging concern in urban stormwater: spatiotemporal patterns and removal by iron-enhanced sand filters (IESFs). Water Res..

[28] Andersson-Sköld Y., Johanesson M., Gustafsson M., Järlskog I., Lithner D., Polukarova M., Strömvall A.-M. (2020). Microplastics from tyre and road wear - a literature review. http://www.vti.se.

[29] Järlskog I., Strömvall A.M., Magnusson K., Galfi H., Björklund K., Polukarova M., Garção R., Markiewicz A., Aronsson M., Gustafsson M., Norin M., Blom L., Andersson-Sköld Y. (2021). Traffic-related microplastic particles, metals, and organic pollutants in an urban area under reconstruction. Sci. Total Environ..

[30] Krotz L., Giazzi G. (2017). Elemental Analysis : CHNS/O characterization of polymers and plastics. https://assets.thermofisher.com/TFS-Assets/CMD/Application-Notes/an-42230-oea-chnso-polymers-plastics-an42230-en.pdf.

[31] (2016). Naturvårdsverket, Rening av avloppsvatten i Sverige. https://www.naturvardsverket.se/Documents/publikationer6400/978-91-620-8808-8.pdf.

[32] Skumlien Furuseth I., Støhle Rødland E. (2020). Reducing the release of microplastic from tire wear: nordic efforts.

[33] Dagle R.A., Winkelman A.D., Ramasamy K.K., Lebarbier Dagle V., Weber R.S. (2020). Ethanol as a renewable building block for fuels and chemicals. Ind. Eng. Chem. Res..

[34] Edenhofer O., Pichs-Madruga R., Sokona Y., Farahani E., Kadner S., Seyboth K., Adler A., Baum I., Brunner S., Eickemeier P., Kriemann B., Savolainen J., Schlömer S., von Stechow C., Zwickel T., Minx J.C. (2014). Climate Change 2014: Mitigation of Climate Change. Contribution of Working Group III to the Fifth Assessment Report of the Intergovernmental Panel on Climate Change.

[35] USEPA (2012). Global anthropogenic non-CO 2 greenhouse gas emissions: 1990-2030. http://www.epa.gov/climatechange/EPAactivities/economics/nonco2projections.html.

[36] (2021). Naturvårdsverket, Territoriella utsläpp och upptag av växthusgaseritle. https://www.naturvardsverket.se/data-och-statistik/klimat/vaxthusgaser-territoriella-utslapp-och-upptag/.

[37] United States Environmental Protection Agency (2023). Greenhouse gas emissions from a typical passenger vehicle. https://www.epa.gov/greenvehicles/greenhouse-gas-emissions-typical-passenger-vehicle.

[38] Wärmark K., Eklund V. (2023). Emissions of greenhouse gases from waste by type of greenhouse gas and subsector. Year 1990 -.

[39] Spekreijse J., Lammens T., Parisi C., Ronzon T., Vis M. (2019). Insights into the European market for bio-based chemicals.

[40] Golovko O., Örn S., Sörengård M., Frieberg K., Nassazzi W., Lai F.Y., Ahrens L. (2021). Occurrence and removal of chemicals of emerging concern in wastewater treatment plants and their impact on receiving water systems. Sci. Total Environ..

[41] Undeman E., Rasmusson K., Kokorite I., Leppänen M.T., Larsen M.M., Pazdro K., Siedlewicz G. (2022). Micropollutants in urban wastewater: large-scale emission estimates and analysis of measured concentrations in the Baltic Sea catchment. Mar. Pollut. Bull..

[42] Mackay D., Wania F. (1995). Transport of contaminants to the Arctic: partitioning, processes and models. Sci. Total Environ..

[43] Yuan B., McLachlan M.S., Roos A.M., Simon M., Strid A., de Wit C.A. (2021). Long-chain chlorinated paraffins have reached the arctic. Environ. Sci. Technol. Lett..

[44] Szidat S., Ruff M., Perron N., Wacker L., Synal H.-A., Hallquist M., Shannigrahi A.S., Yttri K.E., Dye C., Simpson D. (2009). Fossil and non-fossil sources of organic carbon (OC) and elemental carbon (EC) in Göteborg, Sweden. Atmos. Chem. Phys..

[45] Allen S., Allen D., Phoenix V.R., Le Roux G., Durántez Jiménez P., Simonneau A., Binet S., Galop D. (2019). Atmospheric transport and deposition of microplastics in a remote mountain catchment. Nat. Geosci..

[46] Evangeliou N., Grythe H., Klimont Z., Heyes C., Eckhardt S., Lopez-Aparicio S., Stohl A. (2020). Atmospheric transport is a major pathway of microplastics to remote regions. Nat. Commun..

[47] Dris R., Gasperi J., Saad M., Mirande C., Tassin B. (2016). Synthetic fibers in atmospheric fallout: a source of microplastics in the environment?. Mar. Pollut. Bull..

[48] Gewert B., Plassmann M., Sandblom O., Macleod M. (2018). Identification of chain scission products released to water by plastic exposed to ultraviolet light. Environ. Sci. Technol. Lett..

[49] Zhu L., Zhao S., Bittar T.B., Stubbins A., Li D. (2020). Photochemical dissolution of buoyant microplastics to dissolved organic carbon: rates and microbial impacts. J. Hazard Mater..

[50] Ward C.P., Armstrong C.J., Walsh A.N., Jackson J.H., Reddy C.M. (2019). Sunlight converts polystyrene to carbon dioxide and dissolved organic carbon. Environ. Sci. Technol. Lett..

[51] Tang K.W., McGinnis D.F., Ionescu D., Grossart H.P. (2016). Methane production in oxic lake waters potentially increases aquatic methane flux to air. Environ. Sci. Technol. Lett..

[52] Cousins I.T., Dewitt J.C., Glüge J., Goldenman G., Herzke D., Lohmann R., Ng C.A., Scheringer M., Wang Z. (2020). The high persistence of PFAS is sufficient for their management as a chemical class. Environ. Sci. Process. Impacts..

[53] Cousins I.T., Ng C.A., Wang Z., Scheringer M. (2019). Why is high persistence alone a major cause of concern?. Environ. Sci. Process. Impacts..

[54] Macleod M., Breitholtz M., Cousins I.T., De Wit C.A., Persson L.M., Rudén C., McLachlan M.S., Cynthia A., Persson L.M., Rudén C., McLachlan M.S., De Wit C.A., Persson L.M., Rudén C., McLachlan M.S. (2014). Identifying chemicals that are planetary boundary threats. Environ. Sci. Technol..

[55] Kirschbaum M.U.F., Zeng G., Ximenes F., Giltrap D.L., Zeldis J.R. (2019). Towards a more complete quantification of the global carbon cycle. Biogeosciences.

[56] Rochman C.M., Hoellein T. (2020). The global odyssey of plastic pollution. Science.

[57] Kaushal S.S., Wood K.L., Galella J.G., Gion A.M., Haq S., Goodling P.J., Haviland K.A., Reimer J.E., Morel C.J., Wessel B., Nguyen W., Hollingsworth J.W., Mei K., Leal J., Widmer J., Sharif R., Mayer P.M., Newcomer Johnson T.A., Newcomb K.D., Smith E., Belt K.T. (2020). Making ‘chemical cocktails’ – evolution of urban geochemical processes across the periodic table of elements. Appl. Geochemistry.

[58] Wang Z., Walker G.W., Muir D.C.G., Nagatani-Yoshida K. (2020). Toward a global understanding of chemical pollution: a first comprehensive analysis of national and regional chemical inventories. Environ. Sci. Technol..

[59] Fischer S., Almkvist Å., Karlsson E., Åkerblom M. (2006). Preparation of a product register based ExposureIndex. https://www.diva-portal.org/smash/get/diva2:1148159/FULLTEXT01.pdf.

[60] Undeman E., Fischer S., McLachlan M.S. (2011). Evaluation of a novel high throughput screening tool for relative emissions of industrial chemicals used in chemical products. Chemosphere.

[61] Lindquist G., Malmberg A., Sölvell Ö. (2002). Swedish cluster maps: a statistical inventory of clusters in Sweden in 2002. https://www.hhs.se/contentassets/f51b706e1d644e9fa6c4d232abd09e63/swedish_cluster_maps__eng_1.pdf.

[62] Struijs J. (2014). SimpleTreat 4.0: a model to predict fate and emission of chemicals in wastewater treatment plants Background report describing the equations SimpleTreat 4.0: a model to predict the fate and emission of chemicals in wastewater treatment plants Background r. http://www.rivm.nl/en.

[63] Lu L., Guest J.S., Peters C.A., Zhu X., Rau G.H., Ren Z.J. (2018). Wastewater treatment for carbon capture and utilization. Nat. Sustain..

[64] Kätelhön A., Meys R., Deutz S., Suh S., Bardow A. (2019). Climate change mitigation potential of carbon capture and utilization in the chemical industry. Proc. Natl. Acad. Sci. U. S. A..

